# Harmonics management and hosting capacity enhancement: Optimal double-resistor damped double-tuned power filter with artificial hummingbird optimization

**DOI:** 10.1371/journal.pone.0303207

**Published:** 2024-05-10

**Authors:** Mohammed M. Alhaider, Shady H. E. Abdel Aleem, Ziad M. Ali, Ahmed M. Zobaa

**Affiliations:** 1 Department of Electrical Engineering, College of Engineering in Wadi Alddawasir, Prince Sattam Bin Abdulaziz University, Wadi Alddawasir, Saudi Arabia; 2 Department of Electrical Engineering, Institute of Aviation Engineering and Technology, Giza, Egypt; 3 Aswan Faculty of Engineering, Electrical Engineering Department, Aswan University, Aswan, Egypt; 4 Faculty of Engineering, Electrical Power Engineering Department, Cairo University, Giza, Egypt; Graphic Era Deemed to be University, INDIA

## Abstract

This paper introduces a novel and improved double-resistor damped double-tuned passive power filter (DR-DDTF), designed using multi-objective optimization algorithms to mitigate harmonics and increase the hosting capacity of distribution systems with distributed energy resources. Although four different topologies of single-resistor damped double-tuned filters (DDTFs) have been studied before in the literature, the effectiveness of two different DR-DDTF configurations has not been examined. This work redresses this gap by demonstrating that via comprehensive simulations on two power systems, DR-DDTF provides better harmonic suppression and resonance mitigation than single-resistor alternatives. When it comes to optimizing the DR-DDTF for maximum hosting capacity and minimum system active power losses, the multi-objective artificial hummingbird outperformed six other algorithms in the benchmark. To allow for higher penetration of distributed generation without requiring grid upgrades, this newly developed harmonic mitigation filter provides a good alternative.

## 1. Introduction

The integration of renewable energy sources into electric grids has gained significant attention in the past few years because of their environmental benefits and the potential to decrease dependence on fossil fuels [1]. However, the integration of these sources presents various challenges, including the hosting capacity (HC) limitations of the distribution systems. HC refers to the maximum limit of renewable energy that can be safely and reliably accommodated within the existing grid infrastructure. As the penetration of renewable energy sources increases, it becomes crucial to enhance the HC to fully utilize the potential of these clean energy resources [2].

Several factors inherently limit the HC of a distribution system. For instance, the power system’s voltage stability, thermal limits, and protection schemes impose constraints on the maximum limit of renewable energy that can be integrated. Additionally, the presence of harmonics, originating from various sources such as the grid, nonlinear loads, and inverter-based renewable distributed generation, further complicates the HC enhancement process. Harmonics introduce voltage and current distortions, leading to increased losses, reduced equipment lifespan, and decreased system reliability [3].

One promising approach to enhance the HC of harmonically distorted distribution systems is through harmonic mitigation techniques. Harmonic mitigation aims to minimize the adverse effects of harmonics, thereby increasing the system’s capacity to accommodate renewable energy sources. Various mitigation techniques have been proposed, including active power filters, passive filters, and hybrid filter configurations. Among these approaches, passive power filters have gained attention due to their simplicity, low cost, and ease of implementation [4]. According to [5], the cost of passive filters is significantly less than active and hybrid filters for the same rating, especially in high power applications. Shunt-passive and series-passive filters are used to mitigate AC and DC drives harmonics rather than active or hybrid filters due to their performance and cost effectiveness in [6].

Passive power filters employ passive components, such as inductors and capacitors, to attenuate harmonics and improve power quality. These filters can suppress a wide range of harmonic frequencies, making them suitable for HC enhancement. Passive power filter design and optimization for harmonic mitigation within the context of HC enhancement is an ongoing research field in line with the literature [7,8].

The aim of this paper is to propose a novel approach for HC enhancement in harmonically distorted distribution systems using a double-resistor damped double-tuned passive power filter (DR-DDTF). The primary novelty lies in the design of this type of passive power filter, as the damped double-tuned passive power filters (DDTF) have six different schemes presented before in the literature. The four single-resistor schemes of this filter have been investigated before in literature, but the two double-resistors have not been designed, analyzed, or investigated. One more contribution of this paper is the utilization of different multi-objective optimization techniques in the design process to maximize HC and minimize system active power losses simultaneously. By employing six multi-objective optimization algorithms–multi-objective particle swarm optimization (MOPSO), multi-objective slime mould optimization (MOSMA), multi-objective thermal exchange optimization (MOTEO), non-dominated sorting whale optimization (NSWO), non-dominated sorting Harris Hawks optimization (NSHHO), and multi-objective artificial hummingbird algorithm (MOAHA), the performance of the proposed filter is thoroughly evaluated.

To assess the effectiveness of the proposed method, two power systems are considered, each characterized by a different set of harmonic signatures. The first system comprises 30 harmonic signatures, while the second system includes 49 harmonic signatures. The harmonic sources in these systems include the grid, nonlinear loads, and inverter-based renewable distributed generation. Comparative analysis is conducted to evaluate the performance of the proposed filter design using the different optimization algorithms.

The paper is organized as follows: The literature review is presented in Section 2. The system formulation including: the system components, the filter design equations, different power quality indices, the problem formulation, and the used optimization algorithms are presented in Section 3. are presented in Section 4. The results and discussions are presented in Section 4. The conclusions and future work are presented in Section 5.

## 2. Literature review

Mirbagheri S. et al. [9] and Ismael S. et al. [2,10] provided an in-depth definition and explanation of HC, emphasizing its role in accommodating high levels of distributed generation while maintaining grid stability and power quality. The authors also stressed the importance of HC as a critical parameter for integrating renewable energy into the grid and enhancing energy sustainability. The authors in [9] stated that the goal of many researchers was -for so long- the determination of the optimal DG sizing and sitting while neglecting an important practical factor that can make these studies hard to implement. This factor is the highest amount of generation that the distribution grid can accommodate while adhering to grid constraints. It is a crucial performance indicator that must be taken into account when planning and operating the grid. The authors in [2,10] stated that the HC concept was presented to solve the conflict between the DGs owners/investors and the power system operators, as the owners tend to install more and more DGs to maximize their profits, while the system operators are disturbed by the issues and the adverse effects of the excessive DG penetration in the networks.

Researchers like Yuan J. et al. [11] and Qamar N. et al. [12] have investigated the factors that influence HC in power systems. Their study highlighted the impact of voltage limits, thermal limits, and power flow constraints on the HC of distribution networks. Understanding these determinants is crucial for devising effective strategies to improve HC. The authors in [11] determined the maximum HC of photovoltaic (PV) systems in electric grids considering some performance and operating power network indices like the optimal power flow, the network standard voltage limits, the network thermal and ampacity limits, and the power factor limits. The authors in [12] stated the aforementioned performance and operating constraints as in [11] and extended the limiting factors of the HC to voltage imbalance, voltage flicker and harmonics.

HC determination can be achieved using different techniques. According to [2], The assessment of HC is subject to uncertainty, primarily due to the unknown locations of DGs, their various unit ratings, their intermittent nature due to the changing weather conditions, and load profile variations. According to [12], there are various HC determination approaches such as the deterministic approach, the stochastic approach, the time series approach, the worst-case hour approach, and the stochastic time series approach. The main difference between these approaches is the consideration of uncertainty factors, the intermittent operation of DGs, and other practical uncertainty and limiting factors.

**Fig 1:**
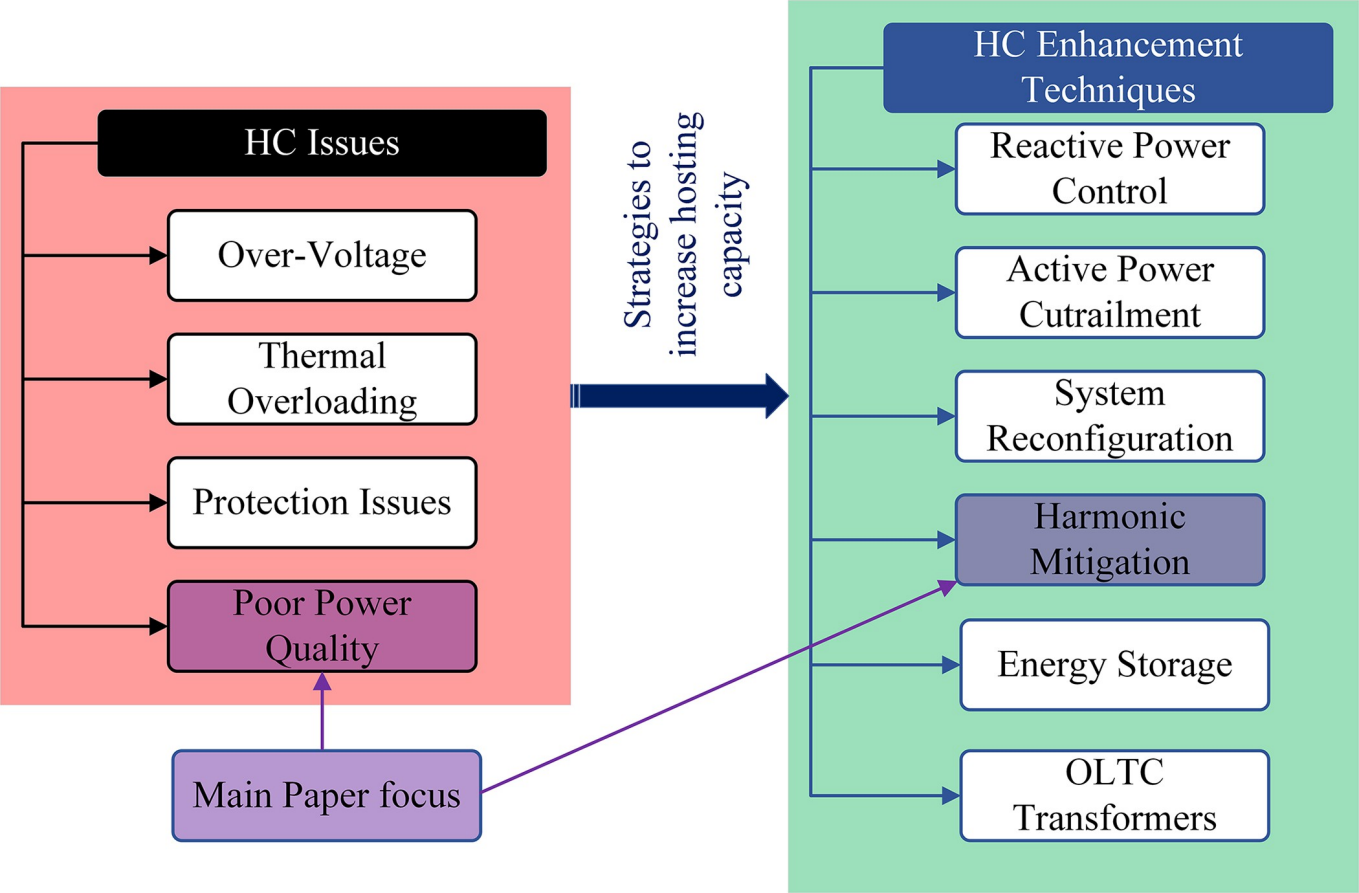
HC issues and their suggested solutions from the literature.

To mitigate these risks, it is essential to carefully design and manage the penetration of RES or distributed generation (DG) into the electrical power system, or in other words, increase the system HC. In the literature, many solutions have been recommended to achieve this goal. These solutions can include implementing advanced control and monitoring systems to manage the power flow dynamically and utilizing energy storage solutions to even out fluctuations in renewable energy output. In addition, using effective harmonic mitigation strategies can help address the issue of harmonic distortion and ensure that the system remains stable and efficient. DG active power reduction, the system reactive power control, and the use of an on-load tap changer (OLTC) are also some of the suggested solutions. Several solutions can be used together to increase HC while maintaining system indices within their standard limits in more complex systems [2,4,10,13–18]. The HC issues, some of the suggested HC enhancement techniques, and the main paper focus are depicted in Fig 1.

Recently, there has been a significant amount of research dedicated to enhancing the HC of distribution networks as an alternative to costly upgrades. This section delves into various methodologies and approaches discussed across a range of literature, all aimed at increasing the HC of electrical networks. The authors in [13] introduced a technique for optimally selecting conductors utilizing the salp swarm optimization (SSO) algorithm. The primary aim was to simultaneously lower the combined annual investment expenses and energy costs, all while adhering to system voltage limits and the thermal/mechanical capacities of the conductors. Despite the outlined strategy having some practical challenges, the same article introduced an alternative method for reinforcing feeders, ultimately resulting in improved HC values.

**Fig 2:**
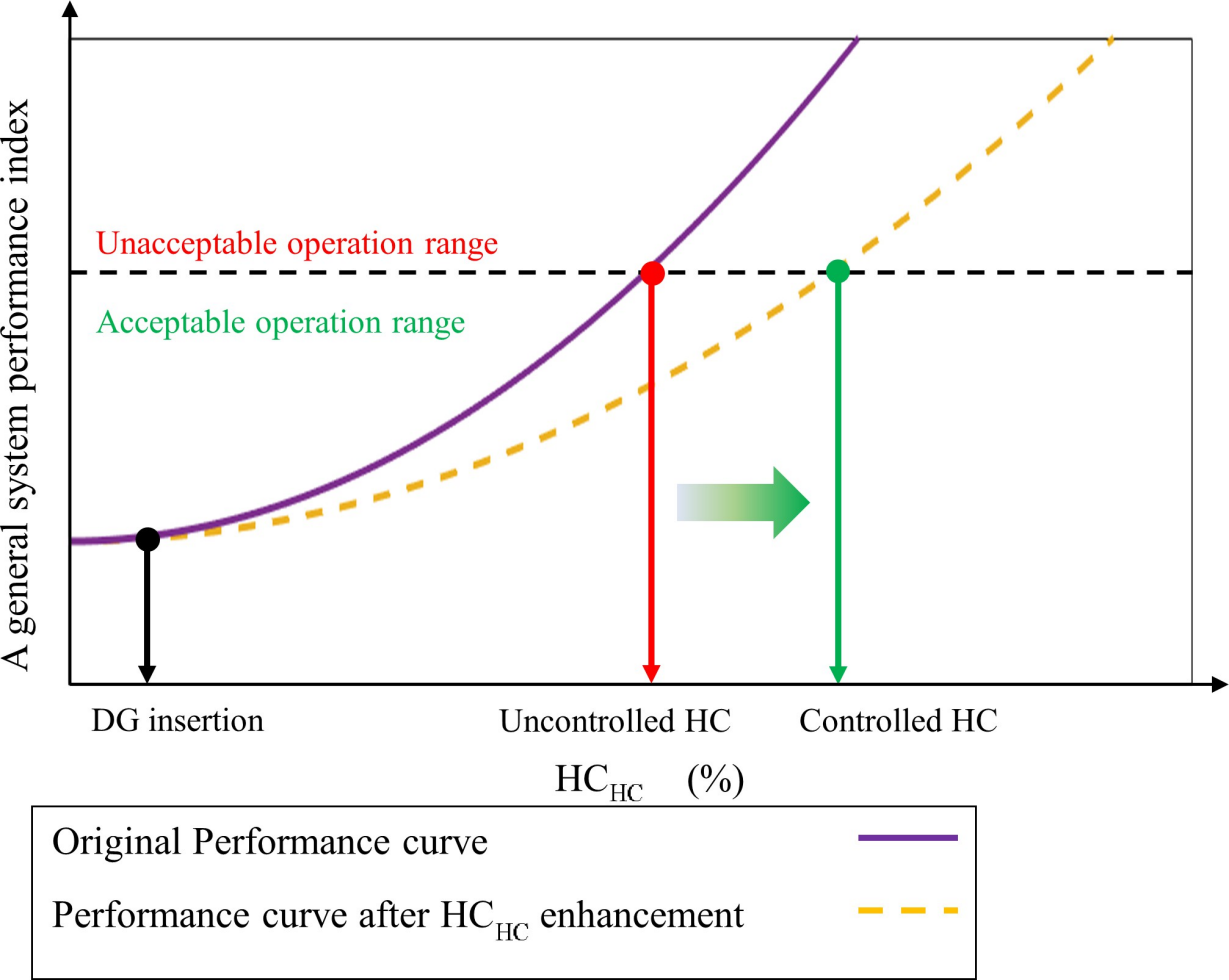
HC enhancement illustration using a general system performance.

Fig 2 illustrates harmonic-constrained HC (HC_HC_) enhancement using a general system performance index, where the objective is to increase the HC while maintaining the index within its acceptable operating range.

In the context of [12], the primary aim was to effectively manage the dynamic fluctuations in voltage within DG-integrated systems. To enhance voltage regulation in both stable and changing conditions, an adaptable controller is suggested for STATCOMs used within low-voltage (LV) grids. This controller contributes to the enhancement of hosting capacity, encompassing both static and dynamic scenarios. In [19], a method involving risk assessment was employed to evaluate the hosting capacity of active distribution networks (ADNs), considering various uncertainties of DGs. A probabilistic approach is utilized to account for system uncertainty. Additionally, a range of techniques has been devised to augment network hosting capacity, including demand response in smart grids, as highlighted in [19,20]. The authors in [21] presented an optimal placement of PV systems, while the authors in [22] introduced two reactive power control techniques, namely cos(φ) and *Q*(*V*), along with prediction-based active power control involving DGs. These control strategies are tested on an existing distribution grid featuring a single medium voltage (MV) feeder and numerous LV grids. The results indicated that employing the *Q*(*V*) strategy can boost the hosting capacity of an area by up to 15% compared to the cos(φ) strategy. The authors in [23] contributed by a voltage control strategy for active transformers, which, according to existing literature, shifts the concern from overvoltage in the network to the ratings of grid components when installing PV systems. However, it was important to note that these techniques are not widely applicable but are dependent on grid topology and specifications and possess certain limitations.

According to [12], the HC enhancement techniques in the literature can be categorized into the following different categories: OLTC transformer employment, curtailment of dispersed generation resources, optimized PV deployment, active power control, reactive power control, network reconfiguration, the use of battery energy storage system, and harmonic mitigation.

Focusing on harmonic mitigation as an HC enhancement technique, harmonics give rise to issues of diminished power quality and energy wastage within the system. Hence, it becomes essential to implement appropriate protective measures against harmonic disturbances prior to embarking on the grid design process. The authors in [4] employed a C-type passive power filter to mitigate harmonics in a distribution system increasing its HC while achieving satisfactory values of different harmonic indices according to the IEEE 519 standards. In [24], the HC evaluation of a two-bus industrial distribution system was carried out, taking harmonic restrictions into account. The research revealed a steep reduction in HC as nonlinearity of the load side increases. To alleviate these harmonics, a passive C-type filter was suggested. This solution not only maximizes HC but also delivers enhanced voltage regulation, improved power factors, and harmonics limitation, leading to an impressive 55.34% increase in HC. In [25], the authors introduced a comprehensive formula capable of determining HC at specific points in distribution and transmission systems affected by harmonic distortions. Applying this equation to an actual power system yielded a HC range of 37 MW for the worst-case scenario and 160 MW for the best-case scenario. Using an alternating current-based optimal power flow method, the authors of [26] addressed harmonics by focusing on voltage and thermal restrictions, which were the main factors limiting HC. In order to determine allowable limitations for DG integration, the authors of [27] carefully aligned the method with acceptable harmonic analysis assumptions for different scenarios and created precise equations. Traditional radial distribution systems can benefit from this method, which is both technically and economically sound.

Among the previous solutions suggested in the literature, harmonic mitigation using power filters increases the HC_HC_ of the system, improves its power factor (both displacement and distortion components), decreases the system active power losses, and improves other system indices such as: increasing the load voltage, and decreasing the source current. Passive power filters are cheaper and more suitable for high-voltage and high-power applications than active power filters [28]. Damped passive power filters are used to avoid harmonic resonance problems while improving the system power quality, as in [7,8].

Harmonic pollution and overloading of different system components are considered as power loss and power quality concerns in this study. They occur because of unplanned addition and sizing of DGs, the use of electronic-based loads, and the rapidly increasing installation of harmonic-injecting RES in electric systems.

Further, the MOAHA is used for maximizing the HC_HC_ and minimizing system active power losses (*P*_*LOSS*_) of a harmonic-polluted electric distribution system. Utility-side voltage harmonics are included, as are current harmonics created by the nonlinear load and generated by the DGs.

A shunt-connected capacitor bank is used to investigate HC enhancement under sinusoidal conditions. A damped double-tuned filter (DDTF) is designed and connected parallel to the load to improve the system’s power quality. There are six schemes for this filter in the literature, four of them are single-resistor schemes, and two of them are double-resistor. Only the single-resistor schemes were designed, analyzed, and compared in the literature; however, one scheme (scheme B) was proven to be superior compared to the others [8]. This filter provides harmonic mitigation of the system, which improves power quality indices such as: voltage total harmonic distortion (*THD*_*V*_), current total demand distortion (*TDD*_*I*_), and individual harmonic voltage and current distortions (*IHD*_*V*_ and *IHD*_*I*_) and corrects the true power factor (*PF*) of a combination of linear and nonlinear loads. The allowable levels of harmonic distortion are determined by following the IEEE 519 guidelines [29]. Limits on encouraged power factor, thermal capacity of transmission lines, and system voltage are all factors in Egypt’s electrical distribution code [30] that are met in the study.

## 3. System formulation

### 3.1. System description and parameters

**Fig 3:**
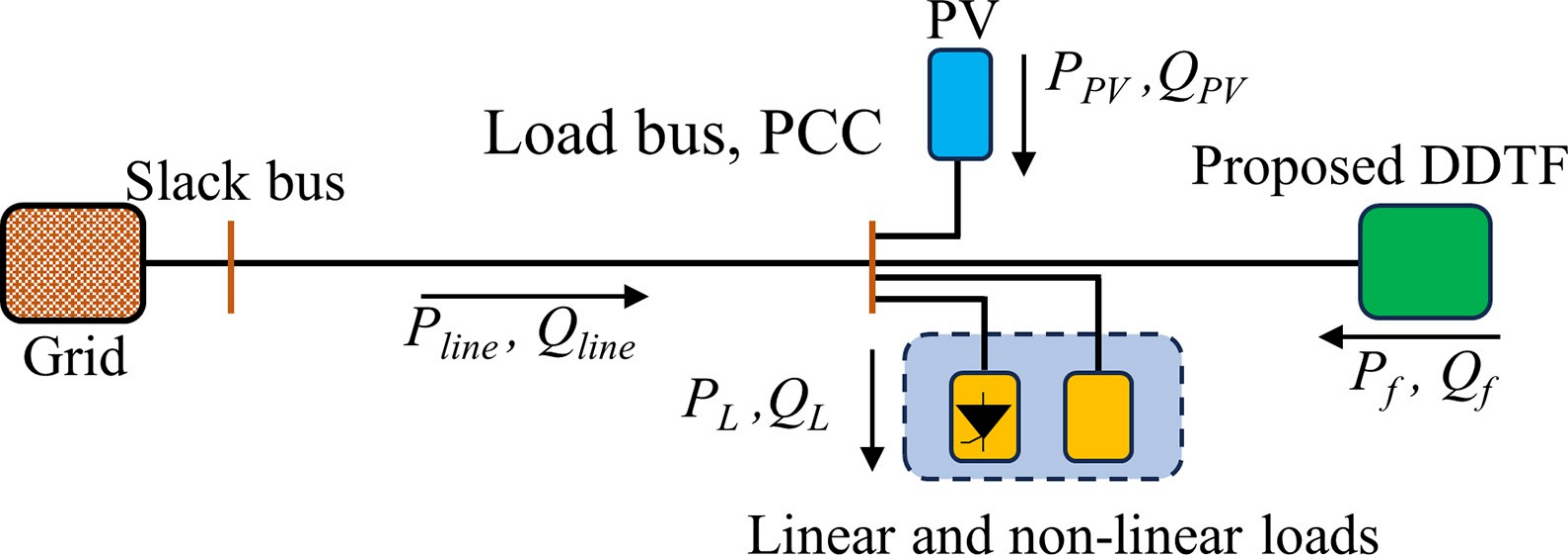
The studied system.

The system consists of a utility grid (reduced to its Thevenin’s equivalent), a PV generator which is an inverter-based harmonic-generating DG source, a combination of linear and non-linear load, and the proposed passive power filter. All the system components are connected to the point of common coupling (PCC). The system is depicted in Fig 3.

Two test system parameters are used in this paper. The values of nonlinear load and PV generator *IHD*_*I*_, Grid-side *IHD*_*V*_ are obtained from the literature to compare the results with those achieved in [4,29]. The first test system (TS_1_) contains 30 harmonic orders, while the second (TS_2_) contains 49 harmonic orders. For both systems, the non-linearity ratio (*NLL*), which is the ratio of the nonlinear load power to the total load power equals 25%. The parameters of TS_1_ are shown in Table 1.

**Table 1:** TS_1_ parameters.

Parameter	Value
Base voltage (kV)	13.8
Base current (A)	314
Base Power (kVA)	7500
Frequency (Hz)	50
Rated load active power (p.u.)	0.92
Rated load reactive power (p.u.)	0.39
Thevenin’s resistance (Ω)	0.0115
Thevenin’s reactance (Ω)	0.1154

The values of *IHD*_*I*_ for the non-linear load (*IHD*_*INLL*_) and *IHD*_*V*_ for the grid-side harmonics (*IHD*_*VS*_), also referred to as background voltage distortion for TS_1_, as well as their maximum limits (*IHD*_*I*,*MAX*_, *IHD*_*V*,*MAX*_) implied by IEEE 519 based on the system voltage and short-circuit capacity with the corresponding harmonic orders (*h*) are shown in Table 2. Also, the values of *IHD*_*I*_ for the PV generator (*IHD*_*IPV*_) used in TS_1_ are shown in Table 3. The parameters of TS_2_ are shown in Table 4. The values of *IHD*_*INLL*_ for TS_2_ and *IHD*_*I*,*MAX*_ implied by IEEE 519 based on the system voltage and short-circuit capacity with the corresponding *h* are shown in Table 5. The values of *IHD*_*VS*_ for TS_2_ and *IHD*_*VS*,*MAX*_ implied by IEEE 519 based on the system voltage and short-circuit capacity with the corresponding *h* are shown in Table 6. The values of *IHD*_*IPV*_ used in TS_2_ are shown in Table 7.

**Table 2:** Non-linear load’s individual harmonic currents and grid’s individual harmonic voltages as percentages of their fundamental values for TS_1_ and their maximum limits implied by IEEE 519.

*h*	Non-linear load		Grid-side harmonics	
	IHD_INLL_ (%)	IHD_I,MAX_ (%)	IHD_VS_ (%)	IHD_V,MAX_ (%)
5	20.00	7.00	3.00	3.00
7	14.30	7.00	2.00	3.00
11	9.10	3.50	2.00	3.00
13	7.70	3.50	1.00	3.00
17	5.90	2.50	1.00	3.00
19	5.30	2.50	1.00	3.00
23	4.30	1.00	1.00	3.00
25	4.00	1.00	0.50	3.00
29	3.40	1.00	0.50	3.00

**Table 3:** The individual harmonic currents of the PV generator used in TS_1_ as a percentage of its fundamental current.

*h*	*IHD*_*IPV*_ (%)	*h*	*IHD*_*IPV*_ (%)	*h*	*IHD*_*IPV*_ (%)
1	100	11	0.67	21	0.50
2	1.13	12	0.80	22	0.40
3	3.27	13	0.46	23	0.20
4	0.26	14	1.06	24	0.35
5	3.48	15	0.30	25	1.33
6	0.12	16	0.50	26	0.19
7	1.12	17	1.48	27	0.61
8	0.82	18	0.59	28	1.20
9	0.49	19	1.14	29	0.90
10	0.84	20	0.71	30	0.67

**Table 4:** TS_2_ parameters.

Parameter	Value
Base voltage (kV)	11
Base current (A)	525
Base Power (kVA)	10000
Frequency (Hz)	50
Rated load active power (p.u.)	0.72
Rated load reactive power (p.u.)	0.38
Thevenin’s resistance (Ω)	0.455
Thevenin’s reactance (Ω)	1.165

**Table 5:** Non-linear load’s individual harmonic currents as a percentage of its fundamental values for TS_2_.

*h*	*IHD*_*INLL*_ (%)	*h*	*IHD*_*INLL*_ (%)	*h*	*IHD*_*INLL*_ (%)
3	15.00	19	5.30	35	2.43
5	12.00	21	4.74	37	2.21
7	11.00	23	4.32	39	2.06
9	8.05	25	4.01	41	1.88
11	7.15	27	3.79	43	1.64
13	6.42	29	3.40	45	1.47
15	5.87	31	2.86	47	1.35
17	5.44	33	2.62	49	1.26

**Table 6:** The grid’s harmonic voltages as percentages of their fundamental values for TS_2_ and their maximum limits implied by IEEE 519.

*h*	*IHD*_*VS*_ (%)	*IHD*_*V*,*MAX*_ (%)	*h*	*IHD*_*VS*_ (%)	*IHD*_*V*,*MAX*_ (%)
*h*<7	2.00	3.00	25 ≤ *h* ≤ 35	0.50	3.00
7 ≤ *h <* 13	1.25	3.00	35 ≤ *h* < 45	0.25	3.00
13 ≤ *h* <25	0.80	3.00	45 ≤ *h* < 49	0.15	3.00

**Table 7:** The individual harmonic currents of the PV generator used in TS_2_ as a percentage of its fundamental current.

*h*	*IHD*_*IPV*_ (%)	*h*	*IHD*_*IPV*_ (%)	*h*	*IHD*_*IPV*_ (%)
2	0.3281	18	0.4344	34	0.0642
3	3.9664	19	1.3261	35	0.2945
4	0.5835	20	0.4288	36	0.0884
5	3.7461	21	1.1262	37	0.2760
6	0.8764	22	0.4066	38	0.0670
7	2.7612	23	0.5980	39	0.2480
8	0.9513	24	0.2670	40	0.0360
9	2.4682	25	0.5322	41	0.2360
10	0.9814	26	0.2150	42	0.0240
11	1.9091	27	0.5030	43	0.2040
12	0.7603	28	0.1970	44	0.0170
13	1.8321	29	0.4850	45	0.1940
14	0.6713	30	0.1690	46	0.0090
15	1.6222	31	0.4240	47	0.1470
16	0.5524	32	0.1120	48	0.0351
17	1.4140	33	0.3970	49	0.1220

### 3.2. Filter design

**Fig 4:**
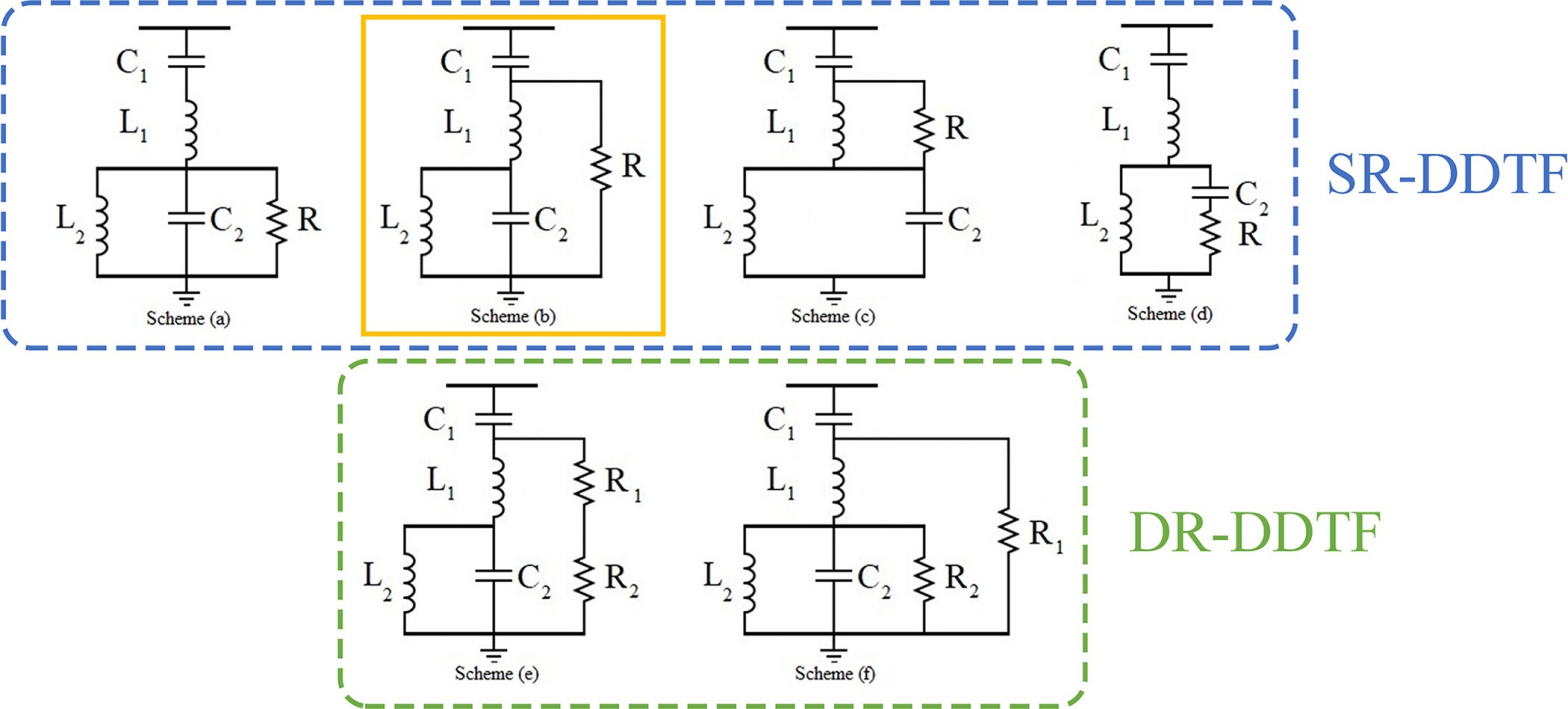
DDTF schemes featuring Scheme (B) as the best SR-DDTF scheme.

The different DDTF schemes are shown in Fig 4. As mentioned earlier, there are six different DDTF schemes; four of them are single-resistor DDTF (SR-DDTF), and two are DR-DDTF. The DR-DDTFs have not been designed nor investigated in literature before. Hence, in this paper, DR-DDTFs are utilized as well as Scheme B as the best SR-DDTF according to [8]. The direct design equations of the undamped double-tuned filter (DTF) design equations were introduced in [29]. A comparison between the different design methodologies of this filter was conducted in [25].

**Fig 5:**
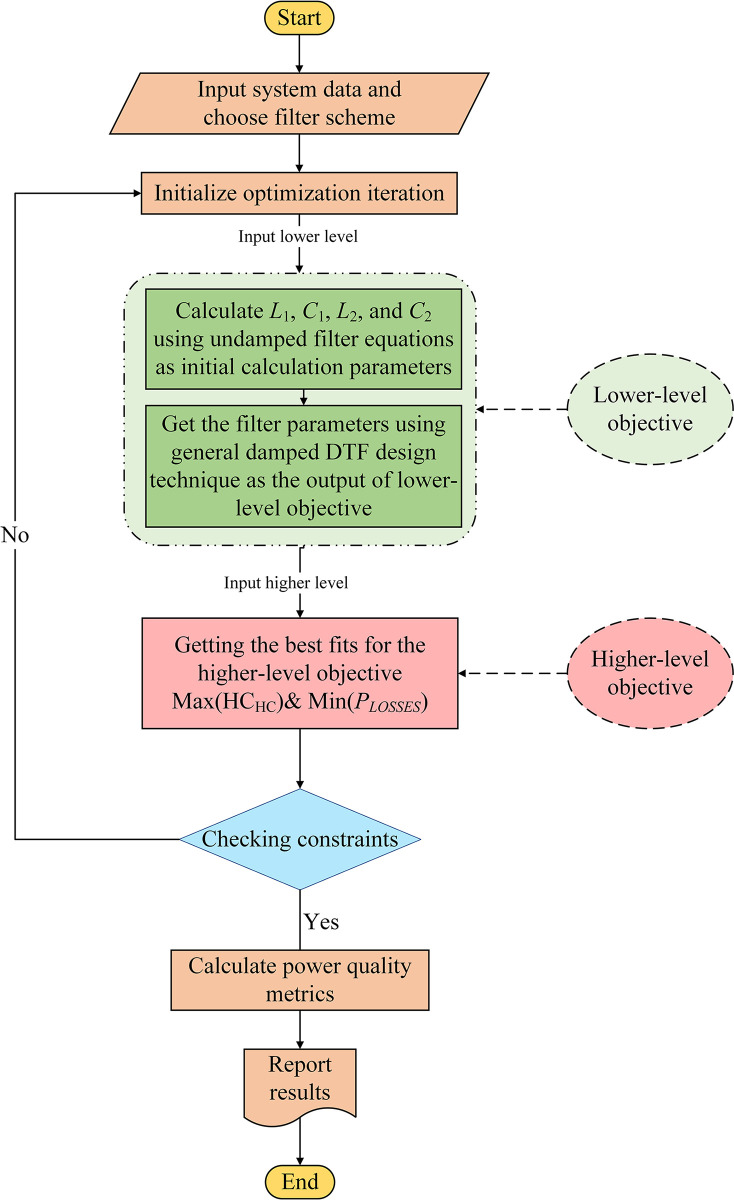
Bi-level multi-objective optimization problem flowchart.

According to [5], DDTFs should be designed using bi-level optimization, where the filter is designed as an undamped DTF first. Then, the lower-level objective function (OF_L_) minimizes the difference between the tuning frequencies of the DTF (*ω*_1_, *ω*_2_) and the frequencies at which the DDTF has the lowest impedance value (*ωz*_1_, *ωz*_2_). The higher-level objective function (*OF*_*H*_) is the system indices that need to be improved; in this case, the maximization of *HC*_*HC*_ and the minimization of *P*_*LOSS*_ simultaneously. Therefore, this problem is tackled using bi-level multi-objective optimization. The flowchart of this optimization problem is shown in Fig 5.

The DTF parameters (*L*_1_,*L*_2_,*C*_1_,*C*_2_) can be obtained by the following equations:

$$C_{1}=\left(\omega_{f}(\frac{\omega_{p}}{\omega_{1}\omega_{2}})‐\frac{1}{\omega_{f}}+\frac{\omega_{f}}{\omega_{1}^{2}\omega_{2}^{2}}[\frac{(\omega_{1}^{2}+\omega_{2}^{2}‐\omega_{p}^{2})\omega_{p}^{2}‐\omega_{1}^{2}\omega_{2}^{2}}{\omega_{p}^{2}‐\omega_{f}^{2}}]\right)\times \left(\frac{Q_{f}}{V^{2}}\right)$$ (1)

$$C_{2}=C_{1}{\left(\frac{{\omega_{1}}^{2}+{\omega_{2}}^{2}‐{\omega_{p}}^{2}}{\omega_{s}^{2}}‐1\right)}^{‐1}$$ (2)

$$L_{1}=\frac{1}{\omega_{s}^{2}C_{1}}$$ (3)

$$L_{2}=\frac{1}{\omega_{p}^{2}C_{2}}$$ (4)

$$\omega_{s}=\frac{1}{\sqrt{L_{1}C_{1}}}$$ (5)

$$\omega_{p}=\frac{1}{\sqrt{L_{2}C_{2}}}$$ (6)

where *ω*_*f*_ is the fundamental angular frequency, *ω*_1_, and *ω*_2_ are the two tuning angular frequencies of the DTF, *ω*_*s*_ is the tuning angular frequency of the series part of the DTF, *ω*_*p*_ is the tuning angular frequency of the parallel part of the DTF, *Q*_*f*_ is the reactive power of the filter in VARs, and *V* is the voltage of the PCC.

### 3.3. System performance indices

The distribution line’s current at *h* (*I*_*Sh*_) can be given as follows:

$$\bar{I_{\mathrm{Sh}}}=\frac{\bar{V_{\mathrm{Sh}}}‐\bar{V_{\mathrm{Lh}}}}{\bar{Z_{\mathrm{Lh}}}}$$ (7)

where *V*_*Sh*_ is the *h*th source (slack-bus) voltage, *V*_*Lh*_ is the *h*th PCC voltage, and *Z*_*Lh*_ is the *h*th distribution line’s impedance which can be given as follows:

$$\bar{Z_{\mathrm{Lh}}}=R_{\mathrm{Lh}}+\mathrm{jh}X_{L}$$ (8)

where *R*_*Lh*_ and *X*_*L*_ are the real and imaginary parts of the line’s equivalent impedance at *h* and at the fundamental frequency, respectively.

The source current *I*_*S*_, the load or PCC voltage *V*_*L*_ and the filter current *I*_*f*_ are obtained by the following equations:

$$I_{S}=\sqrt{\sum_{h\geq 1}I_{\mathrm{Sh}}^{2}}$$ (9)

$$V_{L}=\sqrt{\sum_{h\geq 1}V_{\mathrm{Lh}}^{2}}$$ (10)

$$I_{f}=\sqrt{\sum_{h\geq 1}I_{\mathrm{fh}}^{2}}$$ (11)

where *I*_*fh*_ is the filter current at *h* and is expressed as:

$$I_{\mathrm{fh}}=\frac{\left|V_{\mathrm{Lh}}\right|}{\left|Z_{\mathrm{fh}}\right|}$$ (12)

The harmonic derating factor (*HDF*), which quantifies the overloading of transmission lines in harmonically distorted systems, can be expressed as:

$$HDF=\frac{1}{\sqrt{1+\sum_{h\geq 2}\frac{{I_{Sh}}^{2}\times R_{Lh}}{{I_{S1}}^{2}\times R_{L1}}}}$$ (13)

*P*_*LOSS*_ can be expressed as:

$$P_{\mathrm{LOSS}}=P_{\mathrm{line}}+P_{\mathrm{filter}}$$ (14)

where *P*_*line*_ is the distribution line’s active power losses, and *P*_*filter*_ is the filter’s active power losses that can be determined as follows:

$$P_{line}=\sum_{h\geq 1}\left(I_{sh}^{2}R_{Lh}\right)$$ (15)

The true load power factor (*PF*) and the displacement power factor (*DPF*) at PCC are formulated as:

$$\mathrm{PF}=\frac{\sum_{h\geq 1}\left(V_{\mathrm{Lh}}\times I_{\mathrm{Sh}}\times \cos\left(\phi_{h}\right)\right)}{V_{L}\times I_{S}}\times 100\%$$ (16)

$$\mathrm{DPF}=\frac{V_{L1}\times I_{S1}\times \cos\left(\phi_{1}\right)}{V_{L1}\times I_{S1}}\times 100\%$$ (17)

where *ϕ*_*h*_ is the angle between $\bar{V_{\mathrm{Lh}}}$ and $\bar{I_{\mathrm{Sh}}}$ at *h*, *V*_*L*1_ is the fundamental-frequency load voltage, *I*_*S*1_ represents the fundamental-frequency source current, and *ϕ*_1_ is the angle between $\bar{V_{L1}}$ and $\bar{I_{S1}}$.

The total harmonic distortion for the PCC voltage (*THD*_*V*_) and the total demand distortion for the source current (*TDD*_*I*_) are expressed, respectively, as follows:

$${\mathrm{THD}}_{V}=\frac{\sqrt{\sum_{h\geq 2}V_{\mathrm{Lh}}^{2}}}{V_{L1}}$$ (18)

$${\mathrm{TDD}}_{I}=\frac{\sqrt{\sum_{h\geq 2}I_{\mathrm{Sh}}^{2}}}{I_{\mathrm{LM}}}$$ (19)

where *I*_*LM*_ represents the fundamental-frequency maximum load ampacity. The individual PCC voltage *IHD*_*VL*_ and line current *IHD*_*IL*_ harmonics can be obtained by:

$${\mathrm{IHD}}_{\mathrm{VL}}=\frac{V_{\mathrm{Lh}}}{V_{L1}}\times 100\%$$ (20)

$${\mathrm{IHD}}_{\mathrm{IL}}=\frac{I_{\mathrm{Sh}}}{I_{S1}}\times 100\%$$ (21)

Finally, *HC*_*HC*_ can be calculated as follows:

$$HC_{\mathrm{HC}}=\frac{S_{\mathrm{PV}}}{S_{L\;\mathrm{rated}}}\times 100\%$$ (22)

where *S*_*PV*_ is the apparent power rating of the PV generator, and *S*_*L rated*_ is the apparent power rating of the load.

### 3.4. Problem formulation

#### 3.4.1. Objective functions

For DDTF Scheme B, the *OF*_*L*_ can be expressed as:

$$OF_{L}=\min(\max(\Delta \omega_{z1},\Delta \omega_{\mathrm{z2}}))(Q_{f},L_{1},{C_{1},L}_{2},C_{2},R,h_{1},h_{2},m_{p})$$ (23)

where *Δω*_*Z*1_ = *ω*_*Z*1_−*ω*_1_, and *Δω*_*Z*2_ = *ω*_*Z*2_−*ω*_2_. For both Schemes E and F, the *OF*_*L*_ can be expressed as:

$$OF_{L}=\min(\max\left(\Delta \omega_{z1},\Delta \omega_{\mathrm{z2}}\right))(Q_{f},L_{1},{C_{1},L}_{2},C_{2},R,h_{1},h_{2},m_{p})$$ (24)

The *OF*_*H*_ can be expressed as follows:

$$OF_{H}=\left\{ \begin{array}{c} {\mathrm{OF}}_{1}=Max\;HC_{\mathrm{HC}}(S_{\mathrm{PV}},\emptyset_{\mathrm{PV}})\\ {\mathrm{OF}}_{2}=Min\;P_{\mathrm{LOSS}}(S_{\mathrm{PV}},\emptyset_{\mathrm{PV}}) \end{array}\right.$$ (25)

where ϕ_PV_ is the power angle of the PV generator. So, determining the optimal values of the *S*_*PV*_ and ϕ_PV_ gives *P*_*PV*_ and *Q*_*PV*_, respectively, the PV generator’s active and reactive powers.

#### 3.4.2. Constraints

The previous objective functions are subjected to the following constraints (*Con*):

$${\mathrm{Con}}_{1}={V_{\mathrm{rms}}}_{\min}<{V_{L}}_{\mathrm{rms}}<{V_{\mathrm{rms}}}_{\max}$$ (26)

where ${V_{rms}}_{min}$ and ${V_{rms}}_{max}$ are set to 95% and 105%, respectively.

$${\mathrm{Con}}_{2}={I_{L}}_{\mathrm{rms}}<{I_{\mathrm{Lrms}}}_{\max}$$ (27)

where ${I_{Lrms}}_{max}$ is the maximum distribution line ampacity.

$${\mathrm{Con}}_{3}=\left\{ \begin{array}{c} {\mathrm{DPF}}_{\min}<DPF<{\mathrm{DPF}}_{\max}\\ {\mathrm{PF}}_{\min}<PF<{\mathrm{PF}}_{\max} \end{array}\right.$$ (28)

where *DPF*_*min*_ and *PF*_*min*_ are set to 0.92, and *DPF*_*max*_ and *PF*_*max*_ are set to 1.

$${\mathrm{Con}}_{4}=HC_{\mathrm{HC}}\leq 100$$ (29)

$${\mathrm{Con}}_{5}=\left\{ \begin{array}{c} {\mathrm{THD}}_{V}\leq {{\mathrm{THD}}_{V}}_{\max}\\ IHD_{\mathrm{VL}}\leq {\mathrm{IHD}}_{\mathrm{VLmax}}\\ {\mathrm{TDD}}_{I}\leq {{\mathrm{TDD}}_{I}}_{\max}\\ IHD_{\mathrm{IL}}\leq {\mathrm{IHD}}_{\mathrm{ILmax}} \end{array}\right.$$ (30)

where the maximum limits of the individual and total voltage, and current harmonic distortion are implied by IEEE 519. *THD*_*Vmax*_ equals 5%, and *TDD*_*Imax*_ equals 8%.


$${\mathrm{Con}}_{6}=\left\{ \begin{array}{c} L_{1}>0\\ C_{1}>0\\ L_{2}>0\\ C_{2}>0 \end{array}\right.$$ (31)

### 3.5. Optimization algorithms

The recently developed multi-objective artificial hummingbird algorithm (MOAHA) is a bio-intelligence-based multi-objective optimization technique introduced in 2022 [31]. According to its designer, MOAHA has demonstrated superior performance over commonly used algorithms in the literature for multi-objective problems.

MOAHA takes inspiration from the foraging behaviors of hummingbirds in nature. During the optimization process, MOAHA mimics three key hummingbird foraging tactics: directed foraging towards known flower locations, territorial foraging in productive areas, and migration between habitats. Additionally, these foraging behaviors are designed to simulate three flight capabilities of hummingbirds: axial flight, diagonal flight, and omnidirectional maneuvering. MOAHA also employs a memory-based visitation table, modeling a hummingbird’s ability to remember productive flower locations using experience.

Its developers have validated MOAHA on various multi-objective benchmark functions and real-world engineering optimization problems, demonstrating its effectiveness [31,32]. The specific equations, flowchart, and implementation details are available in these references. Initially, MOAHA requires the pre-definition of four key parameters before optimizing an objective function: the maximum number of iterations/generations, number of populations, number of decision variables/problem dimensions, and archive size for storing non-dominated solutions.

After obtaining a Pareto-optimal front (POF) of solutions using MOAHA, a final selection step is incorporated by using a multi-criteria decision-making method called Technique for Order of Preference by Similarity to Ideal Solution (TOPSIS) [33]. This allows for the identification of a single best solution among the POF based on preference criteria. The incorporation of this intelligent selection method enhances MOAHA’s capabilities to provide both multi-objective exploration and focused recommendation abilities.

In summary, MOAHA provides a powerful swarm intelligence-based multi-objective optimizer that takes inspiration from hummingbirds evolved foraging behaviors and flight dynamics. Its performance and expandability by integrating intelligent solution recommendation methods highlights the promise of bio-inspired algorithms [34].

**Fig 6:**
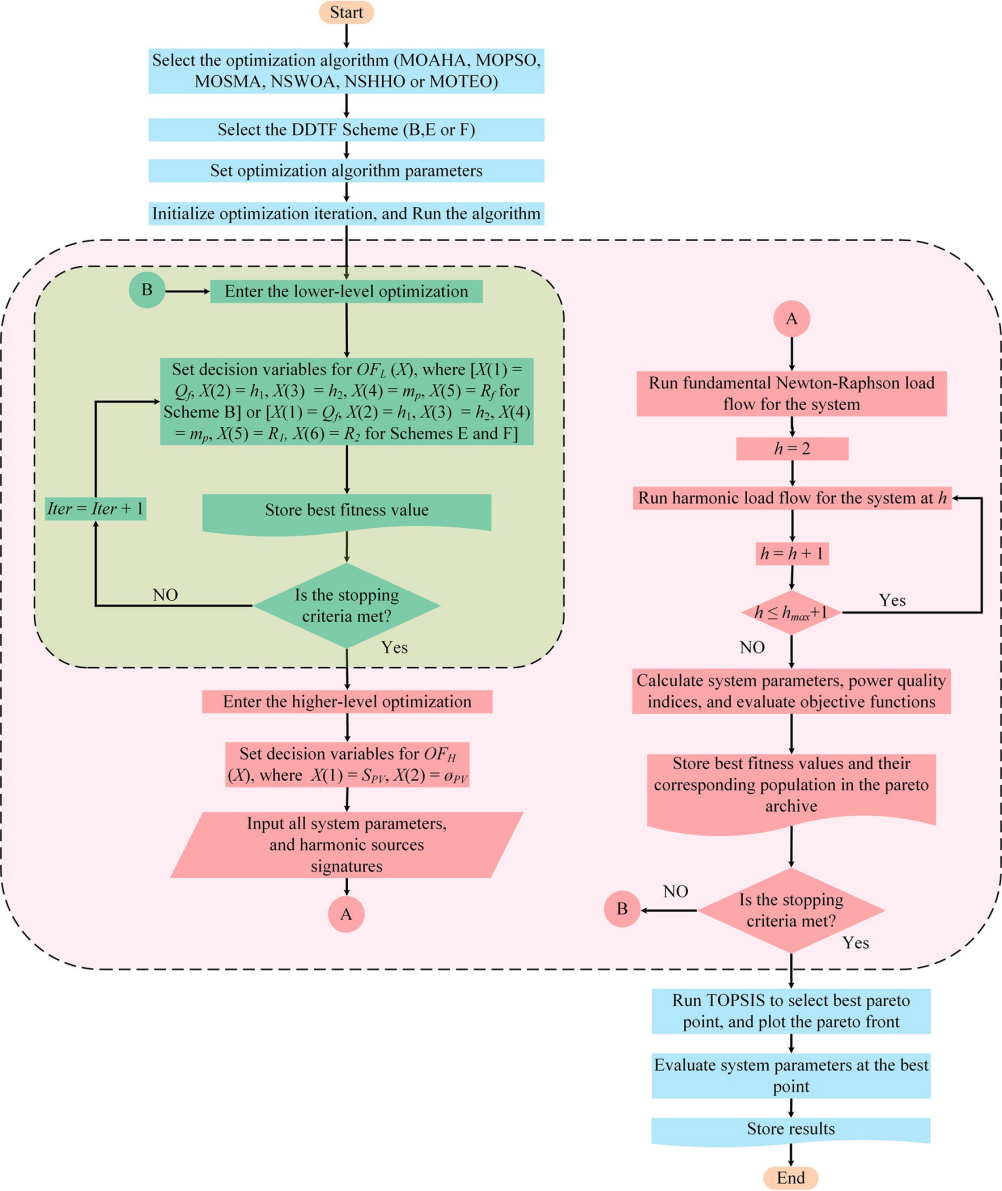
The paper methodology flowchart.

The obtained results from MOAHA are compared to those from five different multi-objective optimization algorithms, namely: the multi-objective particle swarm optimization algorithm (MOPSO) [35], the multi-objective slime mould algorithm (MOSMA) [36], the non-dominated sorting whale optimization algorithm (NSWOA) [37], the non-dominated sorting Harris hawks optimization algorithm (NSHHO) [38], and the multi-objective thermal exchange optimization algorithm (MOTEO) [39] in order to verify the effectiveness and superiority of this technique. The aforementioned methodology is better described by the flowchart in Fig 6.

As shown in Fig 6, *Iter* is the current iteration/generation number, *h*_*max*_ equals 30 for TS_1_ and 49 for TS_2_. The fundamental-frequency optimal power flow for the studied systems is performed using the Newton-Raphson technique to obtain system voltages and currents. Also, a harmonic power flow is carried out at each to get the corresponding harmonic system voltages and currents, and other power quality indices.

## 4. Results and discussions

This section is divided into two sub-sections: one for the results and discussions of TS_1_ and the other for TS_2_. For both systems, the optimization algorithms parameters are shown in Table 8.

**Table 8:** Optimization algorithms parameters.

Optimization Algorithm Parameter	Value
Maximum number of iterations/ generations (*Iter*_*max*_)	500
Number of populations (*N*_*pop*_)	500
Total number of calculations	250,000

### 4.1. TS_1_ results

The fundamental-frequency power flow was used to examine the power quality of the system’s base case and determine the amount of reactive power needed to increase the DPF to its highest value (close to unity). All harmonic signatures from the sources are ignored in this case, only non-linear harmonics are considered. A capacitor bank is connected at the PCC to improve the load *PF*. The capacitor bank is sized so that it supplies all the load reactive power. The simulation results of these cases are shown in Table 9.

**Table 9:** Simulation results of the base and the capacitor bank cases in the absence of sources harmonics for TS_1_.

Parameter	Base Case	Capacitor Bank
Reactive Power (MVAR)	--	2.9250
*V*_*L*_ (p.u.)	0.9484	0.8882
*PF* (%)	91.7991	91.9524
*DPF* (%)	92.0691	92.0691
*HDF* (%)	99.3240	99.2590
*P*_*LOSS*_ (p.u.)	0.0376	0.1710
*TDD*_*I*_ *(%)*	7.0627	7.4204
*THD*_*V*_ *(%)*	3.2076	3.5362
*P*_*PV*_ (MW)	--	6.9000
*Q*_*PV*_ (MVAR)	--	0.0000
*HC* (%)	--	--

In the study undertaken, a capacitor bank was employed to enhance the *PF* of the system, resulting in a near-unity *PF* without the addition of the inverter-based PV generator. However, when the PV generator was introduced into the system, its performance deteriorated, and several key performance metrics exhibited inferior results compared to the base case. The PV generator was added to the system solely for the purpose of supplying active power to the grid, without consuming or supplying reactive power. It should be noted that the analysis focused exclusively on the non-linear load harmonics, disregarding the harmonics from the grid and PV generator.

In the base case scenario, the power factor was found to be below 92%, falling short of the minimum threshold value specified for the PCC voltage (0.95 p.u.). Although the HC theoretically demonstrated a significant improvement, the power factor of the load did not meet the minimum system specifications (below 92%), and the PCC voltage dropped below both the 0.95 p.u. threshold and the corresponding value observed in the base case. Moreover, the presence of the capacitor bank resulted in increased *P*_*LOSS*_ compared to the base case. Additionally, the *THD*_*V*_ and the *TDD*_*I*_ values worsened, albeit still within an acceptable range. Consequently, achieving this desired HC value proved unattainable. The findings of this study highlight the substantial influence that harmonics and power factor values can exert in limiting the penetration levels of DGs, particularly in systems where non-linear load harmonics are prevalent.

When shunt-connected capacitors are employed in distorted systems with harmonics, there is a potential risk of electrical harmonic resonance occurring between the capacitors and the system’s inductive components. This phenomenon can exacerbate the overall operating conditions, leading to adverse effects such as amplified harmonic levels, increased voltage distortion, and even equipment damage. Therefore, the need for effective harmonic mitigation strategies becomes paramount.

To address this challenge, the problem is revisited under conditions of harmonic pollution, considering all three sources of harmonics: the grid, the PV generator, and the non-linear load. By incorporating the harmonic content from all these sources, a comprehensive analysis can be conducted to evaluate the impact on the system performance. This approach enables a more accurate assessment of the harmonic distortion levels, power factor degradation, and voltage quality.

The inclusion of all three harmonics sources allows for a thorough understanding of the system’s behavior and aids in the development of appropriate mitigation techniques. By identifying the dominant harmonic frequencies and their interactions with the shunt-connected capacitors, effective measures can be implemented to suppress resonance and minimize the adverse effects of harmonic distortion. These measures may include the use of harmonic filters, passive or active damping techniques, or the adjustment of capacitor parameters to avoid resonance conditions. The simulation results of the base case, the capacitor bank, and the three DDTF schemes designed using MOAHA are shown in Table 10.

**Table 10:** Simulation results of the base case, the capacitor bank, and the three MOAHA-designed DDTF schemes for TS_1_ with the presence of all system harmonic signatures.

Parameter	Base Case	Compensated System			
		Capacitor Bank	DDTF Scheme B	DDTF Scheme E	DDTF Scheme F
Reactive Power (MVAR)	--	2.9250	5.8616	5.6355	5.4156
*V*_*L*_ (p.u.)	0.9484	0.8890	0.9957	0.9941	0.9933
*PF* (%)	91.7991	91.9501	92.0011	92.0030	92.8689
*HDF* (%)	99.3240	99.4560	99. 4718	99.4561	99.2076
*P*_*line*_ *(kW)*	282	1282	5.9915	10.5202	12.1086
*P*_*filter*_ *(kW)*	--	--	14.3007	0.0005	0.0045
*P*_*LOSS*_ (kW)	282	1282	20.2922	10.5207	12.1131
*TDD*_*I*_ *(%)*	7.0627	6.3769	6.1757	7.6865	7.8223
*THD*_*V*_ *(%)*	3.2076	5.4327	3.6600	3.4595	3.4106
*P*_*PV*_ (MW)	--	6.9000	5.1478	5.7919	5.6787
*Q*_*PV*_ (MVAR)	--	0.0000	1.6871	1.8504	1.6644
*HC*_*HC*_ (%)	--	--	72.2289	81.0709	78.9011

DDTF Scheme E is the best scheme that maximizes *HC* and minimizes *P*_*LOSS*_ simultaneously for TS_1_. The results of Scheme B are better than what is reported in the literature. According to [4], the best *HC*_*HC*_ for this system achieved using C-type passive power filter was found to be 69.86%. SR-DDTF scheme B achieves *HC*_*HC*_ 72.23%, and the two DR-DDTF schemes achieve better values where the best value achieved by Scheme E is about 81.07%. The designed filters parameters for TS_1_ are shown in Table 11.

**Table 11:** The MOAHA-designed DDTFs parameters for TS_1_.

Parameter	DDTF Scheme B	DDTF Scheme E	DDTF Scheme F
*h*_1_	5.0339	2.9935	3.0034
*h*_*2*_	7.3309	5.8834	21.3034
*m*_*p*_	6.8710	4.7633	11.3722
*R*_*f*_ (Ω)	1.7171	--	--
*R*_1_ (kΩ)	--	31.2107	51.1886
*R*_2_ (kΩ)	--	75.3758	5.9393
*C*_1_ (μF)	754.949	746.1905	689.2059
*L*_1_ (μH)	465.2674	993.1815	123.5466
*C*_2_ (μF)	7197.5	1413.8379	55.9159
*L*_2_ (μH)	29.8181	315.8559	1401.1185

### 4.2. TS_2_ results

The base and the capacitor bank cases in the absence of source harmonics for TS_2_ are given in Table 12.

**Table 12:** Simulation results of the base and the capacitor bank cases in the absence of sources harmonics for TS_2_.

Parameter	Base Case	Capacitor Bank
Reactive Power (MVAR)	--	3.8000
*V*_*L*_ (p.u.)	0.95651	0.90744
*PF* (%)	88.1849	88.3415
*DPF* (%)	88.4385	88.4385
*HDF* (%)	99.5320	99.4920
*P*_*LOSS*_ (p.u.)	0.024547	0.10872
*TDD*_*I*_ *(%)*	5.8385	6.096
*THD*_*V*_ *(%)*	2.7071	2.9479
*P*_*PV*_ (MW)	--	7.4888
*Q*_*PV*_ (MVAR)	--	0.0000
*HC* (%)	--	--

For the base case, the *PF* is less than 92%. Trying to improve the system performance using capacitor bank, the *PF* value is still under 92%, and *TDD*_*I*_ value increased compared to the base case due to the existence of the PV generator harmonics.

The simulation results of the base case, capacitor bank case, and the three different DDTF schemes (B, F, and E) designed using MOAHA are shown in Table 13. It can be noticed that in the base, and the capacitor bank cases, the *V*_*L*_, *PF*, and *HDF* values are less (worse) due to the existence of all system harmonics (grid and non-linear load harmonics are added here). The values of *P*_*LOSS*_, *TDD*_*I*_, and *THD*_*V*_ are greater (worse) than the previous case. DDTF Scheme E gives the highest *HC* and the lowest *P*_*LOSS*_ for TS_2_. The value in the capacitor case is greater than 5% which is the maximum limit implied by IEEE 519–2014. For the same system parameters, the highest *HC*_*HC*_ value in the literature was 83.29% achieved by utilizing a hybrid active filtering technique in [40] compared to 76.0796% achieved by DR-DDTF scheme E.

**Table 13:** Simulation results of the base case, the capacitor bank, and the three MOAHA-designed DDTF schemes for TS_2_ with the presence of all system harmonic signatures.

Parameter	Base Case	Compensated System			
		Capacitor Bank	DDTF Scheme B	DDTF Scheme E	DDTF Scheme F
Reactive Power (MVAR)	--	3.8000	5.5033	5.6939	5.5789
*V*_*L*_ (p.u.)	0.9469	0.8835	0.9939	0.9967	0.9937
*PF* (%)	88.1362	88.4385	92.0322	93.7036	92.0226
*HDF* (%)	99.3630	99.5170	99.4584	99.4689	99.4427
*P*_*line*_ *(kW)*	378.61	1733.6	10.6533	3.2667	11.8420
*P*_*filter*_ *(kW)*	--	--	10.6508	4.2636	4.4745
*P*_*LOSS*_ (kW)	378.61	1733.6	21.3041	7.5303	16.3164
*TDD*_*I*_ *(%)*	6.6486	6.081	7.6072	7.9998	7.9453
*THD*_*V*_ *(%)*	5.4902	5.3683	3.4917	3.1787	3.4089
*P*_*PV*_ (MW)	--	7.4888	5.7334	5.8954	5.6496
*Q*_*PV*_ (MVAR)	--	0.0000	1.8009	1.8964	1.8488
*HC*_*HC*_ (%)	--	--	73.8272	76.0796	73.0277

MOAHA was utilized in [41] to design SR-DDTF (scheme B) using TS_1_ data and an *HC*_*HC*_ value of 72.2% was achieved. The designed filters parameters for TS_2_ are shown in Table 14.

**Table 14:** The MOAHA-designed DDTFs parameters for TS_2_.

Parameter	DDTF Scheme B	DDTF Scheme E	DDTF Scheme F
*h*_1_	4.0106	3.5784	2.9803
*h*_*2*_	28.1139	48.6777	20.7403
*m*_*p*_	12.5613	15.5402	15.0016
*R*_*f*_ (Ω)	1.8273	--	--
*R*_1_ (kΩ)	--	33.2724	3.6917
*R*_2_ (kΩ)	--	5.5037	90.9565
*C*_1_ (μF)	758.6649	719.5288	767.9090
*L*_1_ (μH)	165.7546	112.0770	777.1757
*C*_2_ (μF)	107.5961	44.8610	66.1718
*L*_2_ (μH)	596.8126	935.2326	680.3839

**Fig 7:**
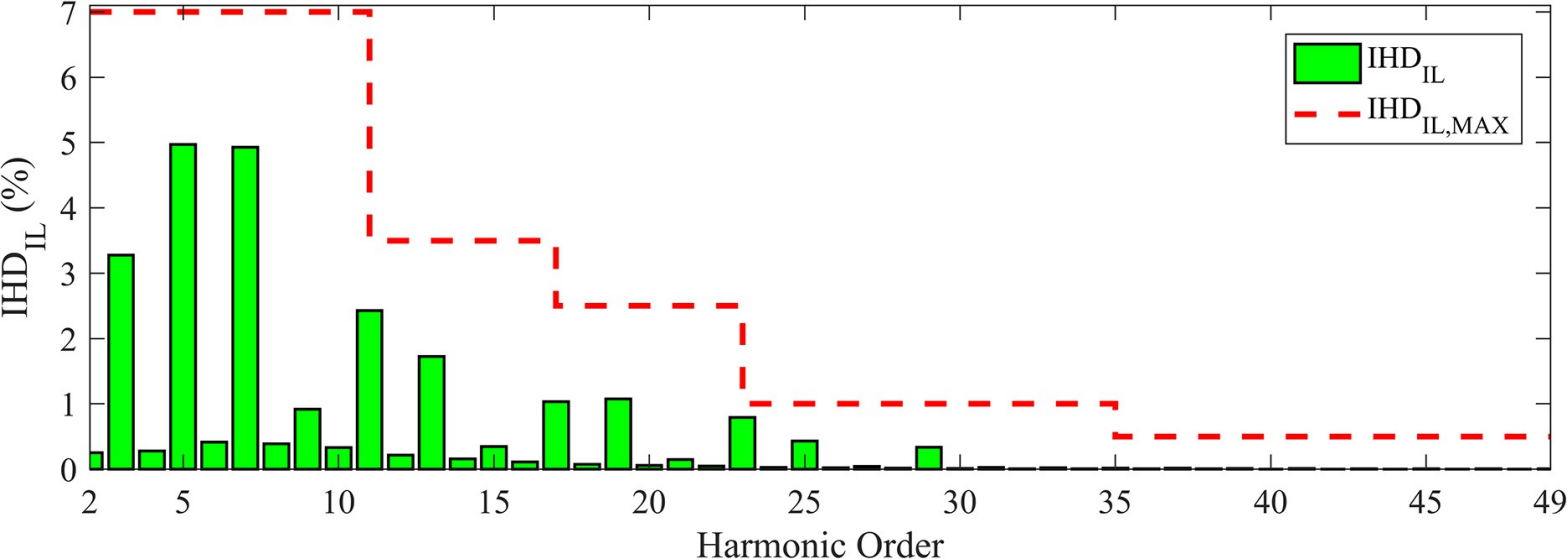
*IHD*_*IL*_ values and the corresponding maximum limits for TS_2_.

The *IHD*_*IL*_ for TS_2_ is depicted in Fig 7.

**Fig 8:**
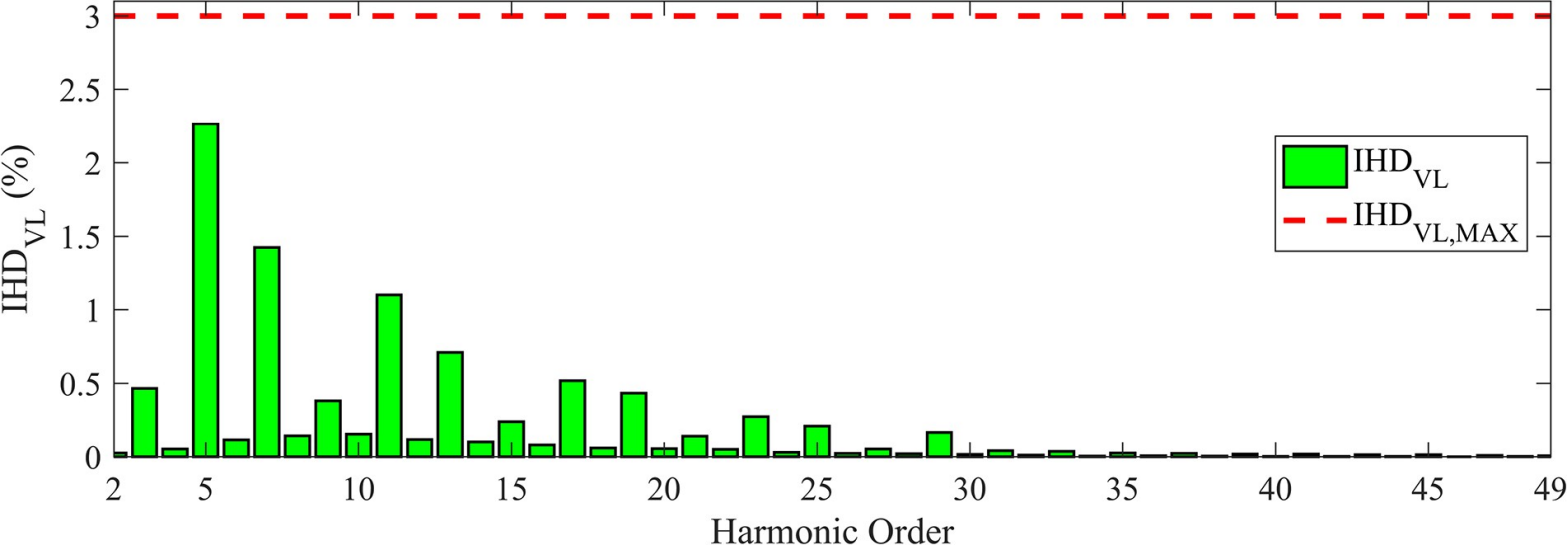
*IHD*_*VL*_ values and the corresponding maximum limits for TS_2_.

The *IHD*_*VL*_ for TS_2_ is depicted in Fig 8.

The values of *IHD*_*IL*_ and *IHD*_*VL*_ are clearly within limits. A comparison between different DDTFs (Scheme E) designed using MOAHA, MOPSO, MOSMA, NSWOA, NSHHO, and MOTEO for TS_*2*_ is shown in Table 15. These results prove the superiority and effectiveness of MOAHA-designed DDTF Scheme E compared to the results obtained using the other algorithms.

**Table 15:** Comparison between system parameters with different DDTFs Scheme E designed using: MOAHA, MOPSO, MOSMA, NSWOA, NSHHO, MOTEO for TS_2_.

Parameter	MOAHA	MOPSO	MOSMA	NSWOA	NSHHO	MOTEO
Reactive Power (MVAR)	5.6939	5.5810	4.7134	5.0083	3.9510	4.5340
*V*_*L*_ (p.u.)	0.9967	0.9936	0.9870	0.9929	0.9833	0.9951
*PF* (%)	93.7036	92.1612	97.7019	93.7037	98.3801	90.3298
*HDF* (%)	99.4689	99.1746	99.2319	99.1576	99.2208	99.1114
*P*_*line*_ *(kW)*	3.2667	12.2166	41.5231	15.4011	65.0720	10.3213
*P*_*filter*_ *(kW)*	4.2636	2.5941	0.2727	0.1837	1.3849	0.3329
*P*_*LOSS*_ (kW)	7.5303	14.8107	41.7958	15.5849	66.4570	10.6543
*TDD*_*I*_ *(%)*	7.9998	7.9802	7.6466	7.9998	7.5773	8.1245
*THD*_*V*_ *(%)*	3.1787	3.3818	3.9298	3.7826	4.6566	4.8165
*P*_*PV*_ (MW)	5.8954	5.6221	4.1404	5.3991	3.3613	4.7749
*Q*_*PV*_ (MVAR)	1.8964	1.8441	1.0472	1.2475	0.3960	0.3461
*HC*_*HC*_ (%)	76.0796	72.6880	52.4669	68.0752	41.5788	58.8140

**Fig 9:**
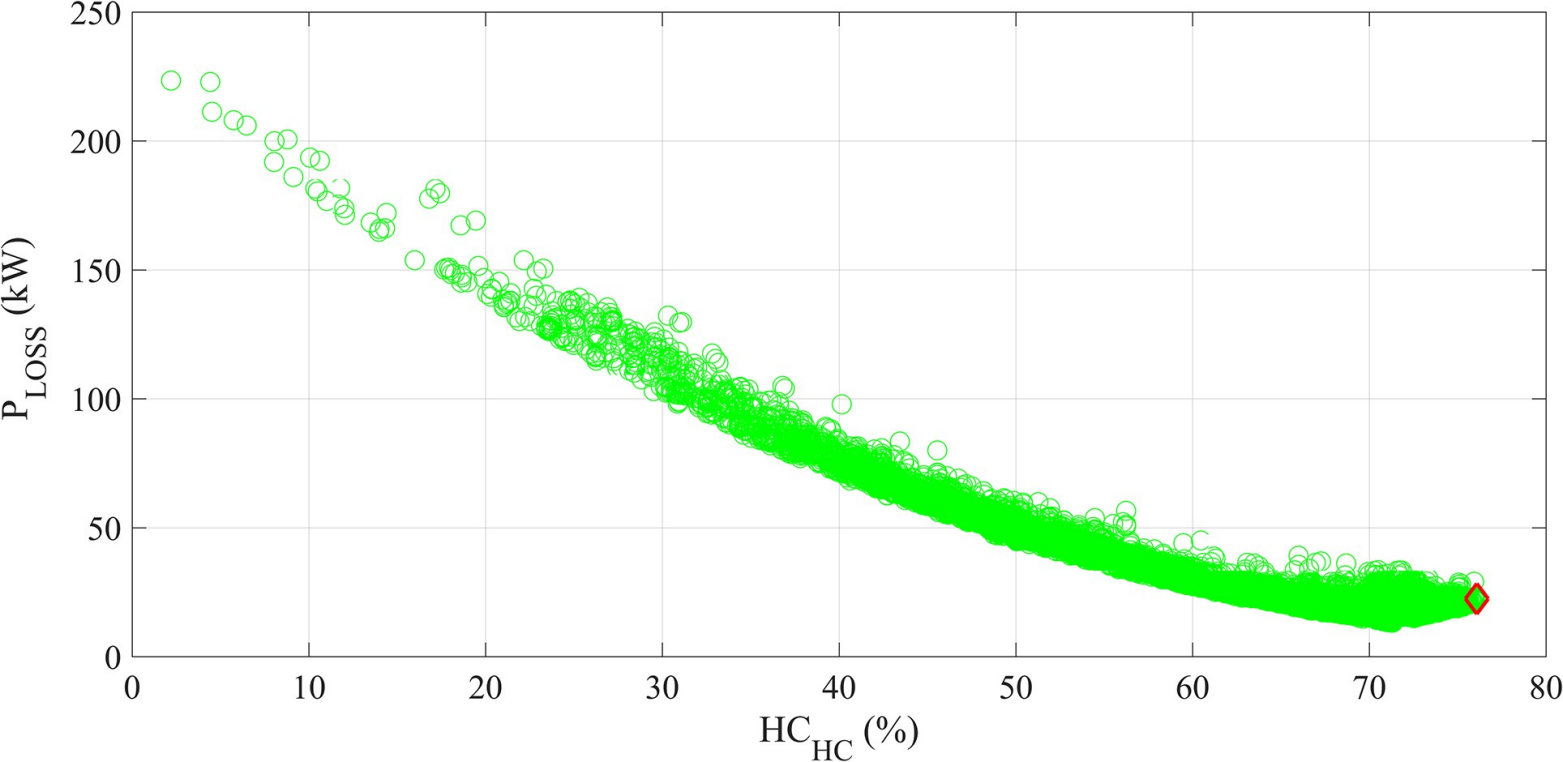
The POF of the solutions for MOAHA-designed DR-DDTF Scheme E for TS_2_.

The POF of the solutions obtained by MOAHA-designed DR-DDTF Scheme E for TS_2_ is shown in Fig 9.

The best solution is to be chosen using TOPSIS, in which the two objectives are given equal weights in this study.

It is clear that MOTEO fails to satisfy the *TDD*_*I*_ and *PF* constraints. The highest *HC*_*HC*_ as well as the lowest *P*_*LOSS*_ values are obtained by MOAHA.

## 5. Conclusions and future work

To sum up, the paper conclusions are discussed in the following bullets:

- In this study, a novel design for the double-resistor damped double-tuned passive power filter (DR-DDTF) was proposed to enhance the hosting capacity while minimizing active power losses in two test systems (TS_1_ and TS_2_).
- The performance of two DR-DDTF schemes (Schemes E and F) was compared with Scheme B, which was previously identified as the best single-resistor DDTF (SR-DDTF) in literature.
- The results obtained from the simulations demonstrated that Scheme E outperformed the other schemes and proved to be the most efficient DR-DDTF for both TS_1_ and TS_2_. This scheme achieved a significant improvement in the hosting capacity while effectively reducing the active power losses while satisfying other system constraints for *V*_*L*_, *PF*, *THD*_*V*_, *TDD*_*I*_, *HDF*, *IHD*_*V*_, and *IHD*_*I*_.
- These findings highlight the superiority of the proposed DR-DDTF design in comparison to the existing SR-DDTF schemes.
- Furthermore, the filters were designed using a bi-level MOAHA. The optimization algorithm was compared to five other optimization techniques, including MOPSO, MOSMA, NSWOA, NSHHO, and MOTEO. The results indicated that MOAHA exhibited superior performance in terms of optimizing the objective functions and achieving the desired filter design.
- Finally, this study presents a significant advancement in the design of passive power filters for enhancing hosting capacity and minimizing active power losses. The use of DR-DDTFs, particularly Scheme E, offers improved performance compared to the existing SR-DDTF scheme. Moreover, the successful application of the bi-level multi-objective artificial hummingbird optimization algorithm demonstrates its effectiveness in designing optimal damped double-tuned filter configurations.

Future work may include further investigations of DR-DDTF Schemes: Although Scheme E has shown superior performance in the tested scenarios, it is essential to explore the potential of other DR-DDTF schemes and evaluate their effectiveness in different system configurations and operating conditions. Comparative studies can shed light on the strengths and limitations of various DR-DDTF designs.

Further techno-economic analysis and assessment may be conducted to evaluate the performance of the proposed DR-DDTFs under uncertainties and variations in system parameters, load profiles, and renewable energy generation which may provide a more comprehensive understanding of their applicability and effectiveness in real-world scenarios.

## Supporting information

S1 File (PDF)
