# Supplementary material for: Harmonics management and hosting capacity enhancement: Optimal double-resistor damped double-tuned power filter with artificial hummingbird optimization
Source: PLoS One. 2024 May 10;19(5):e0303207. doi: 10.1371/journal.pone.0303207 (PMC11086883; doi:10.1371/journal.pone.0303207)
Supplement: S1 File — (PDF) [file pone.0303207.s001.pdf]

```
%System data
basemva = 1;
accuracy = 0.0001;
maxiter = 100;
basekV=13.8;
zbasef=basekV^2/basemva;

% Load_level = 0.7944;   % light
Load_level = 1.0;       % medium
% Load_level = 1.2997;   % heavy                    % It shows the loading level 0; zero load
and 1; maximum load

nll_ratio = 0.25;
thdv_ratio = 0.972;
RNLL = nll_ratio ;
RLL=1 - RNLL;                                                    % load non linear part 25% &
linear part 75%
Vsub=1.0;                                                    % substation voltage
Cable_l=1;                                                   % cable length = 2.4*Cable_l (km)
angle_DG=0;                                                   % DG'nin harmonik açýsý
angle_NLL=0;                                                  % DG'nin harmonik açýsý

%No PV in the base case
%PDG = SDG * cosd(Phi_inv);  % active power of DG
%QDG = SDG * sind(Phi_inv);  % reactive power of DG

%     busdata=[1   1    Vsub     0.0      0                  0          0       0           0    100    0
%                    2   0    1.0      0.0     6.2*Load_level    4.2*Load_level     0      0        -10    10
0];     % Bus data
busdata=[1   1    Vsub     0.0      0                  0          0       0           0    100    0
               2   0    1.0      0.0     6.9*Load_level    2.925*Load_level     0      0        -10    10
0];     % Bus data
linedata=[1   2   0.0337*Cable_l   0.0459*Cable_l    0     1];                    % Linedata

Lfybus;                                                          % form the bus admittance
matrix
Lfnewton;                                                        % Power flow solution by
Newton-Raphson method @1st frequecny

INPV=zeros(1,30);
% No nonlinear load distortion in the base case
INLL=(1/100)*[100*exp(j*angle_NLL)      0*exp(j*angle_NLL)       0*exp(j*angle_NLL)
0*exp(j*angle_NLL)   20*exp(j*angle_NLL)   0*exp(j*angle_NLL)   14.3*exp(j*angle_NLL)
```

```
0*exp(j*angle_NLL)   0*exp(j*angle_NLL)   0*exp(j*angle_NLL)   9.1*exp(j*angle_NLL)
0*exp(j*angle_NLL) 7.7*exp(j*angle_NLL)...
          0*exp(j*angle_NLL)          0*exp(j*angle_NLL)          0*exp(j*angle_NLL)
5.9*exp(j*angle_NLL)    0*exp(j*angle_NLL)  5.3*exp(j*angle_NLL)  0*exp(j*angle_NLL)
0*exp(j*angle_NLL)    0*exp(j*angle_NLL)    4.3*exp(j*angle_NLL)  0*exp(j*angle_NLL)
4*exp(j*angle_NLL)   0*exp(j*angle_NLL)    0*exp(j*angle_NLL)    0*exp(j*angle_NLL)
3.4*exp(j*angle_NLL) 0*exp(j*angle_NLL)];                        % Non- linear load
current spectrum six- pulse 2 type

  VNLL=(1/100)* thdv_ratio *[0 0 0   0  3*exp(j*-pi)  0  2*exp(j*0)   0   0  0  2*exp(j*-pi)
0  1*exp(j*0)...
          0   0  0 1*exp(j*-pi) 0 1*exp(j*0) 0   0   0   1*exp(j*-pi) 0   0.5*exp(j*0) 0   0  0
0.5*exp(j*-pi) 0];                          % Grid side voltage distortion

  h=[1:30];                           % harmonic numbers

  I=zeros(size(h));   VB=zeros(size(h));
  VS=zeros(size(h));  PLS=zeros(size(h));
  PLD=zeros(size(h)); DFCB=zeros(size(h));

  %%-----------------------------------------------------------
  %%-----------------------------------------------------------

  for f=2:30;  % harmonic power flow

    PdpuLL=RLL*Pd(2)/basemva;          % pu real power of load linear part
    QdpuLL=RLL*Qd(2)/basemva;          % pu reactive poower of load linear part
    Qgl = busdata(2,8);
    PdpuNLL=RNLL*Pd(2)/basemva;        % pu real power of load non-linear part
    QdpuNLL=RNLL*Qd(2)/basemva;        % pu reactive power of load non-linear part
    Qgl_inj = busdata(2,11);          % added caps or inductor
    Qgpu_inj=Qgl_inj /basemva;         % per unit value of caps or ind


    Qgpu = Qgl/basemva;                % PFC capacitor power
    Pgpu=Pg(2)/basemva;                % pu real power of gen
    VSH(f)=V(1)*VNLL(f);               % RMS Non linear source voltage
    VSHP(f)=abs(V(1)*VNLL(f)) * sqrt(2); % Peak Non linear source voltage
    IL1=abs((V(1)-V(2))/Z);
    ILp1=(V(1)-V(2))/Z;

    XCAPF = (Vm(2)^2/Qgpu);

    INL(f)=abs(conj((PdpuNLL+j*QdpuNLL)/Vm(2)))*INLL(f);       % NLL load currrent
    IPV(f)=(Pgpu/V(2))*INPV(f);                               % PV harmonic currents
```

```
YL(f)=PdpuLL/(abs(V(2))^2)-j*QdpuLL/(h(f)*(abs(V(2)^2)));   % Load admittance

% No filter in the base case

YFD(f)=1/(R+j*h(f)*X);             % Feeder admittance

YFc_inj(f) = h(f)*j*Qgpu_inj/(Vm(2)^2);  % injected reactive power for 11.

YH(f)=YFD(f)+YL(f)+YFc_inj(f);       % admittance matrice filter admittance will be
added YF
IH(f)=VSH(f)*YFD(f)-INL(f)-IPV(f); % Current Matrix
VBH(f)=IH(f)*inv(YH(f));          % voltage equation [V]=[I]*inv[Y]
ILH(f)=(VSH(f)-VBH(f))*YFD(f);      % Line Current Ih
ILHM(f)=abs(ILH(f));               % hth. Line current rms
VBHM(f)=abs(VBH(f));               % hth. load bus voltage rms
VSHM(f)=abs(VSH(f));               % hth. source bus voltage rms

PFh(f)=real(VBH(f)*ILH(f));        % hth. real power Vh*Ih*cos(phi);
DFCB(f)=(((R*sqrt(h(f)))/R)*((ILHM(f)/1)^2))+DFCB(f); % cable derating determine
% No filter in the base case
%DPf(f)=abs(real(VBH(f)*conj(VBH(f)*YF(f))));              %hth. filter loss in pu
I(f)=ILHM(f)^2+I(f);               % sum of hth. line currents
VB(f)=VBHM(f)^2+VB(f);
VS(f)=VSHM(f)^2+VS(f);

PLD(f)=VBHM(f)*ILHM(f)*cos(angle(VBH(f))-angle(ILH(f))); % hth. real power at load
bus
QLD(f)=VBHM(f)*ILHM(f)*sin(angle(VBH(f))-angle(ILH(f)));  % hth. imag power at
load bus
PLS(f)=(ILHM(f)^2)*real(inv(YFD(f)));                  % hth. Line loss

end

% calculation of parameters

THDV=100*sqrt(sum(VB))/abs(V(2));        % THDV calculation
HDF=((1+sum(DFCB))^(-0.5));              % HDF calculation
VBRMS=sqrt(sum(VB)+(abs(V(2))^2));       % pu load bus rms voltage
ILRMS=sqrt(sum(I)+(IL1^2));              % pu line current rms value
VSRMS=sqrt(sum(VS)+(abs(V(1))^2));       % pu Utility source voltage rms
PLOAD=sum(PLD)+Pd(2)/basemva;            % pu load power
QLOAD=sum(QLD)+Q(2);
PF=100*((abs(V(2))*IL1*cos(angle(V(2))-angle(ILp1)))+sum(PLD))/(VBRMS*ILRMS);
% Power factor at load bus (0-100)
```

```
PFM=1/PF;                          % to find maximum PF
DPF=100*(abs(V(2))*IL1*cos(angle(V(2))-angle(ILp1)))/(abs(V(2))*IL1);                    %
displacement power factor at load bus
PLINELOSS=(IL1^2)*R+sum(PLS);           % pu power loss at line
%No filter in the base case
%PFILTERLOSS=sum(DPf);                 % filter losses
DV=abs(100*(1-VBRMS));
QLOADh=sum(QLD);
QT1=Q(2);

% No penetration level in the base case
%PLev=1/(PDG/7.5);                     % penetration level estimation

% calculation of individual harmonic load voltage
V2H=100*VBHM(2);       V3H=100*VBHM(3);         V4H=100*VBHM(4);
V5H = 100 * VBHM(5);   V6H=100*VBHM(6);         V7H=100*VBHM(7);
V8H=100*VBHM(8);       V9H=100*VBHM(9);         V10H=100*VBHM(10);
V11H=100*VBHM(11);     V12H=100*VBHM(12);       V13H=100*VBHM(13);
V14H=100*VBHM(14);     V15H=100*VBHM(15);       V16H=100*VBHM(16);
V17H=100*VBHM(17);     V18H=100*VBHM(18);       V19H=100*VBHM(19);
V20H=100*VBHM(20);     V21H=100*VBHM(21);       V22H=100*VBHM(22);
V23H=100*VBHM(23);     V24H=100*VBHM(24);       V25H=100*VBHM(25);
V26H=100*VBHM(26);     V27H=100*VBHM(27);       V28H=100*VBHM(28);
V29H=100*VBHM(29);     V30H=100*VBHM(30);

% calculation of individual harmonic line current
ihc2=100*(ILHM(2)/1);   ihc3=100*(ILHM(3)/1);   ihc4=100*(ILHM(4)/1);
ihc5=100*(ILHM(5)/1);   ihc6=100*(ILHM(6)/1);   ihc7=100*(ILHM(7)/1);
ihc8=100*(ILHM(8)/1);   ihc9=100*(ILHM(9)/1);   ihc10=100*(ILHM(10)/1);
ihc11=100*(ILHM(11)/1); ihc12=100*(ILHM(12)/1); ihc13=100*(ILHM(13)/1);
ihc14=100*(ILHM(14)/1); ihc15=100*(ILHM(15)/1); ihc16=100*(ILHM(16)/1);
ihc17=100*(ILHM(17)/1); ihc18=100*(ILHM(18)/1); ihc19=100*(ILHM(19)/1);
ihc20=100*(ILHM(20)/1); ihc21=100*(ILHM(21)/1); ihc22=100*(ILHM(22)/1);
ihc23=100*(ILHM(23)/1); ihc24=100*(ILHM(24)/1); ihc25=100*(ILHM(25)/1);
ihc26=100*(ILHM(26)/1); ihc27=100*(ILHM(27)/1); ihc28=100*(ILHM(28)/1);
ihc29=100*(ILHM(29)/1); ihc30=100*(ILHM(30)/1);

TDD=100*sqrt(sum(I))/1;     % TDD calculation


  YL=PdpuLL/(Vm(2)^2)-j*QdpuLL/(V(2)^2);      % Linear Load admittance
  YNL = PdpuNLL/(Vm(2)^2)-j*QdpuNLL/(V(2)^2); % nonlinear load admittance
```

```
ZLoad = inv(YL);  % load impedance
ZNLoad= inv(YNL);  % nonlinear load impedance
RLoad=real(ZLoad);    RNLoad = real(ZNLoad);
XLoad=imag(ZLoad);    XNLoad = imag(ZNLoad);

INLR = abs(conj((PdpuNLL+j*QdpuNLL)/1));  % nonlinear current
INangle=angle(conj((PdpuNLL+j*QdpuNLL)/V(2)));
IR_peak = INLR*sqrt(2);
IR_angle_rad = INangle;

% Displaying results

disp(['The pu load voltage equals    ',num2str(VBRMS)]);
disp(['The total load PF equals    ',num2str(PF)]);
disp(['The load displacement PF equals    ',num2str(DPF)]);
disp(['The pu line losses equals    ',num2str(PLINELOSS)]);
disp(['The current total demand distortion equals    ',num2str(TDD)]);
disp(['The voltage total harmonic distortion equals    ',num2str(THDV)]);
```
